# Incorporating Post-Cessation Weight-Control Coaching into Smoking Cessation Therapy to Reduce Type 2 Diabetes Risk

**DOI:** 10.3390/nu13103360

**Published:** 2021-09-25

**Authors:** Chien-Hsieh Chiang, Yi-Han Sheu, Fei-Ran Guo, Wan-Wan Lin, Guan-Ru Chen, Kuo-Chin Huang

**Affiliations:** 1 Department of Family Medicine, National Taiwan University Hospital & College of Medicine, Taipei 100, Taiwan; jiansie@ntu.edu.tw (C.-H.C.); fjguo1@ntu.edu.tw (F.-R.G.); 2 Department of Community and Family Medicine, National Taiwan University Hospital Yunlin Branch, Douliu 640, Taiwan; circlechen1982@gmail.com; 3 Graduate Institute of Pharmacology, National Taiwan University College of Medicine, Taipei 100, Taiwan; wwllaura1119@ntu.edu.tw; 4 Taiwan Society for Sports Nutrition, Taipei 111, Taiwan; 5 Department of Psychiatry, Harvard Medical School, Boston, MA 02115, USA; yis828@mail.harvard.edu; 6 Department of Community and Family Medicine, National Taiwan University Hospital Bei-Hu Branch, Taipei 108, Taiwan

**Keywords:** prediabetes, primary care, smoking cessation, type 2 diabetes mellitus, obesity

## Abstract

Post-cessation weight gain (PCWG) facilitates short-term type 2 diabetes (T2D) risk in prediabetic smokers in the absence of complementary measures. In this shared decision-making-based non-randomized controlled trial, prediabetic smokers joined the Fight Tobacco and Stay Fit (FIT2) program or received usual care. The 16-week FIT2 program combined smoking cessation therapy with individualized coaching in diet and physical activity strategies for PCWG restriction (NCT01926041 at ClinicalTrials.gov). During a mean follow-up period of 1316 days, 217 participants (36.8%) developed T2D, and 68 (11.5%) regressed to normoglycemia. In the intention-to-treat analysis (*n* = 589), the FIT2 program was associated with a reduced T2D risk (HR, 0.58; 95% CI, 0.40–0.84) and a higher probability of regression to normoglycemia (HR, 1.91; 95% CI, 1.04–3.53) compared with usual care. The post-program quitters were at lower T2D risk (HR, 0.63; 95% CI, 0.44–0.92) and were more likely to regress to normoglycemia (HR, 1.83; 95% CI, 1.01–3.30) compared with the controls in the time-varying analysis (*n* = 532). We demonstrated that the FIT2 program was negatively associated with long-term T2D risk and positively associated with the probability of regression to normoglycemia compared with usual care. To prevent T2D development, we recommend simultaneously promoting smoking abstinence and lifestyle coaching for PCWG restriction.

## 1. Introduction

Type 2 diabetes (T2D) has been linked to cardiovascular comorbidities, malignancy, and high mortality [1,2,3]. To prevent the occurrence of T2D, lifestyle modification is crucial for individuals with prediabetes [4,5,6]. Given that smoking is also a modifiable lifestyle risk factor for incident T2D [4], smoking cessation may improve insulin resistance through the normalization of insulin receptor substrate-1 phosphorylation at Ser636 [7]. However, studies conducted for non-diabetic smokers showed that smoking cessation in the absence of complementary measures seems to facilitate T2D development [8,9,10,11]. An early cohort study involving non-diabetic middle-aged adults reported that quitters had the highest risk of T2D in the first three years [11]. Findings from another 9.2-year cohort of 2070 overweight Japanese men indicated that T2D occurrence peaked within three years of quitting [10]. A pooled analysis of data from three cohorts in the US further demonstrated that the risk of T2D was not increased among quitters without weight gain [9]. Obviously, post-cessation weight gain (PCWG) restriction could not be manipulated by investigators in these observational studies. Notably, limited literature was focused on prediabetic smokers regarding the link of smoking cessation with T2D development. Multifactorial behavior intervention including intensive lifestyle changes in diet and physical activity to maintain a healthy weight is the cornerstone of diabetes prevention [5,12]. The intervention combining smoking cessation and PCWG restriction has been proposed to minimize obesity and diabetes but has not yet been experimentally elucidated for prediabetic smokers [13,14]. In this non-randomized controlled trial, we introduced a shared decision-making-based Fight Tobacco and Stay Fit (FIT2) program combining smoking cessation therapy with individualized lifestyle coaching for restricting PCWG in prediabetic smokers. The objective of this study was to investigate whether long-term glycemic outcomes would be improved by the FIT2 program and the post-program abstinence, respectively.

## 2. Materials and Methods

### 2.1. Participants

We recruited prediabetic smokers receiving medical care at two hospitals in northern and central Taiwan, where a systematic identification system was applied to classify the tobacco addiction status of every patient in the outpatient clinics [15]. This study invited identified current smokers to undergo screening tests between August 2013 and January 2017. Included were participants with prediabetes who were aged 30 to 75 years and who had smoked ≥10 cigarettes per day for at least six months. Prediabetic participants were those who had repeated results for any of the following parameters: (1) plasma glucose levels 5.6–6.9 mmol/L (100–125 mg/dL) in the fasting state; (2) plasma glucose levels 7.8–11.0 mmol/L (140–199 mg/dL) two hours after a 75 g oral glucose load; and (3) glycated hemoglobin (HbA1c) levels 39–46 mmol/mol (5.7–6.4%), in the absence of antidiabetic drugs [16]. Excluded were those with a pre-existing diagnosis of T2D; thyroid disease; an acute cardiac condition within three months; acute renal failure; chronic glomerulonephritis; polycystic kidney disease; decompensated liver disease; a mental health disorder diagnosed by psychiatrists; malignancy; alcohol consumption exceeding 70 g per week for women or 140 g per week for men; women who were pregnant or breast-feeding; those currently taking antidiabetic drugs, smoking cessation medications, steroids, lithium or antipsychotics. Information about tobacco use, alcohol consumption, physical activity, depression, sleep quality, personal medical histories, and current medications was collected through standardized personal interviews and medical records. The study protocol was approved by the National Taiwan University Hospital Research Ethics Committee (No. 201303041RINB). All participants provided written informed consent. This study was conducted according to the Declaration of Helsinki and was registered at ClinicalTrials.gov (NCT01926041).

### 2.2. Sample Size Estimation

We estimated to recruit at least 596 prediabetic smokers, 33% (199) of whom decided to join the FIT2 program, to reach 90% power and a two-sided 95% CI for the detection of a 50% risk reduction, assuming 30% to be the risk of incident T2D during follow-up in the usual care group [12].

### 2.3. Anthropometric Indices and Laboratory Tests

Body height and weight were measured using a standard stadiometer, and body mass index was calculated. Blood pressure was measured with an electronic sphygmomanometer while the patient was seated and after resting for at least five minutes. Hypertension was defined as a history of hypertension according to the medical records or repeated systolic blood pressure ≥ 140 mmHg or a diastolic blood pressure ≥ 90 mmHg. Each participant underwent laboratory testing after fasting for at least ten hours. Plasma glucose and HbA_1c_ levels, lipids, alanine aminotransferase, and creatinine levels were determined. The estimated glomerular filtration rate was calculated using the four-variable version of the Modification of Diet in Renal Disease Study equation for Chinese Patients [17]. Dyslipidemia was defined as the presence of at least one of the following conditions: a plasma triglyceride level ≥ 1.70 mmol/L (150 mg/dL); a serum low-density lipoprotein level ≥ 3.37 mmol/L (130 mg/dL); a serum high-density lipoprotein level < 1.04 mmol/L (40 mg/dL) in men or <1.29 mmol/L (50 mg/dL) in women; or a history of dyslipidemia according to medical records.

### 2.4. Assignment, Prospective Follow-Up, and Analytic Design

The assignment of this trial was based on shared decision making. At baseline (from August 2013 to January 2017), all eligible prediabetic smokers were asked if they would like to join the FIT2 program or just receive usual care. From enrollment, the smoking status, breath carbon monoxide levels, anthropometric indices, and blood tests were recorded every six months. The intention-to-treat analysis was performed among all participants joining the FIT2 program and receiving usual care. To reduce the immortal time bias in the survival analyses, the time-varying analysis was adopted among the participants with documented abstinence status at six months (Figure S1).

### 2.5. The FIT2 Program and Post-Program Abstinence

The FIT2 program was a 16-week program that combined pharmacotherapy for smoking cessation with individualized counseling focused on both smoking cessation and weight-control techniques. Behavioral counseling in tobacco cessation techniques were delivered to every FIT2 participant involving one session per week [18]. The pharmacotherapy in the FIT2 program was a 16-week varenicline course, which conforms to real-practice government regulations in Taiwan. The varenicline was subsidized by the tobacco health welfare surcharge and its prescription adhered to the manufacturer’s directions and regulations of the Health Promotion Administration, Ministry of Health and Welfare, Taiwan (www.hpa.gov.tw, accessed 19 March 2021). It was initiated at 0.5 mg once daily for the first three days, increased to 1 mg once daily from day 4 to day 7, and then increased to 1 mg varenicline twice daily from day 8 to the end of the 16 weeks. After 16 weeks, varenicline was no longer administered. During the therapy course, the physicians could adjust the varenicline dosages according to tolerability. Participants joining the FIT2 program were encouraged to either set their quit day 8 days after starting the varenicline; or freely choose their quit day for any time between days 8 and 35. Nicotine replacement therapies were prohibited during the study period.

In addition to varenicline prescription and smoking cessation counseling covered in conventional smoking cessation services, the FIT2 program also offered individualized weekly coaching in diet and physical activity to restrict PCWG. The counseling sessions allowed opportunities to identify obstacles to lifestyle change and discuss approaches with a professional panel of dietitians and certified personal trainers. The dietitians instructed the FIT2 participants to choose minimally processed, whole grains, vegetables, whole fruits, nuts, healthful sources of protein, and plant oils; rather than sugared beverages, refined grains, potatoes, red and processed meats, and other highly processed foods. The FIT2 participants were also encouraged to do at least 150 min of moderate-intensity (3.0–6.0 metabolic equivalents) aerobic physical activity throughout the week. Emphasis was placed on checking the weekly diary for body weight, food, and physical activity through protected cellphone messages between the participants and the assigned panel professionals. For those who gained weight, more intensive ways of calorie restriction and physical activity (at least 300 min of moderate-intensity aerobic physical activity per week) were instructed. 

Post-program abstinence status was recorded weekly from 16 weeks to 6 months by self-reported 7-day point prevalence of abstinence and a breath carbon monoxide level of less than 6 ppm. Post-program quitters were those who successfully quit at 16 weeks after the FIT2 program and had maintained their non-smoking status until six months (achieving post-program abstinence). For the time-varying analysis, participants who failed to stop smoking after the FIT2 program were reassigned to the control group, while those who relapsed after six months were still classified as post-program quitters.

### 2.6. Usual Care and Control

Usual care was provided for prediabetic smokers who decided not to join the FIT2 program. Usual care comprised an interpretation of laboratory results and encouragement to quit smoking and initiate a therapeutic lifestyle change for T2D prevention at each visit. In the time-varying analysis, the control group contained participants joining the FIT2 program who failed to achieve post-program abstinence and all participants receiving usual care, including those who quit smoking on their own. Given that the post-program abstinence was established at six months after enrollment, for the time-varying survival analysis, the follow-up time experienced by the controls also included the first six months experienced by all post-program quitters to reduce immortal time bias (Figure S1).

### 2.7. Outcome Measures

The primary outcome was new-onset T2D, defined as random plasma glucose ≥ 11.1 mmol/L (200 mg/dL) with hyperglycemic symptoms or repeated results in at least one of the following parameters: (1) plasma glucose levels ≥ 7.0 mmol/L (126 mg/dL) in the fasting state; (2) plasma glucose levels ≥ 11.1 mmol/L (200 mg/dL) two hours after a 75 g oral glucose load; (3) HbA_1c_ levels ≥ 48 mmol/mol (6.5%) [16]; or currently taking medications for physician-diagnosed T2D. The secondary outcomes included regression to normoglycemia during the study period as well as HbA_1c_ changes at 6 and 12 months and. Participants who regressed to normoglycemia should meet all the following conditions for more than six months and maintain such status until end of the study: (1) plasma glucose levels < 5.6 mmol/L (100 mg/dL) in the fasting state; (2) plasma glucose levels < 7.8 mmol/L (140 mg/dL) two hours after a 75 g oral glucose load; or (3) HbA_1c_ levels < 39 mmol/mol (5.7%), in the absence of antidiabetic drugs. This study expects to collect other preregistered secondary outcomes until December 2022.

### 2.8. Covariates and Confounders

We collected the parameters of the physical activity level, depression scale score, and sleep quality at baseline. The physical activity level within the last week was calculated as the average daily energy expenditure in Kcal/day [19]. Being physically active was determined as the energy expenditure being over the third quartile. The depression score for one week was obtained using the Centre for Epidemiologic Studies Depression Scale. Depressive disorder was suspected if the score ≥ 16, and the patient was referred to a psychiatrist. The sleep quality score for one month was obtained using the Pittsburgh Sleep Quality Index. Sleep disturbance was considered if the score was six or higher.

### 2.9. Statistical Analysis

For the descriptive analyses, values are presented as either numbers (percentages) or mean ± standard deviations. For the univariate analyses, categorical data were compared by the χ^2^ test or Fisher’s exact test. Continuous variables were compared using the two-sample Student’s *t*-test. Statistical significance levels were determined by two-tailed tests (*p <* 0.05). Linear regression analyses were used to estimate the association of clinical variables with HbA_1c_ changes at 6 and 12 months compared with the baseline. We performed Cox proportional hazards regression to explore the association of the FIT2 program with progression to T2D and regression to normoglycemia in the intention-to-treat analysis. Kaplan–Meier failure plots of T2D risks of the two groups were drawn. To reduce immortal time bias, we also adopted time-varying analyses in Cox proportional hazards regression and all participants were considered unexposed from baseline until six months when the post-program abstinence was established [20]. Modified Kaplan–Meier failure plots of the T2D risks by different post-program abstinence statuses at six months were constructed [20]. We assumed missing values over time were missing at random and performed listwise deletion. All of the aforementioned statistical analyses were performed with SAS software version 9.4 (SAS Institute Inc., Cary, NC, USA).

## 3. Results

### 3.1. General Characteristics

A total of 589 eligible prediabetic smokers were enrolled. Among the participants participating in the FIT2 program (*n* = 279), 207 (74.2%) achieved post-program abstinence at six months (Figure S1). Eight participants in the usual care group quit on their own during the follow-up period. At baseline, the age, sex, average cigarette consumption, smoking duration, Fagerström test for nicotine dependence scores, body mass index, HbA_1c_, fasting glucose, alanine aminotransferase, estimated glomerular filtration rate; and the prevalence of hypertension, dyslipidemia, being physically active, depression, and sleep disturbance were comparable either between the FIT2 and the usual care participants in the intention-to-treat analysis (Table S1) or between the post-program quitters and the controls in the time-varying analysis (Table S2). The post-program quitters had greater weight gain at six months compared with the controls (1.26 vs. 0.41 ± 1.28, *p <* 0.001). At the end of this study, there were no serious adverse events reported.

### 3.2. HbA1c Changes

In the intention-to-treat analysis (*n* = 589), weight gain at six months was positively associated with HbA_1c_ changes at 6 and 12 months (β = 0.24 ± 0.05 and 0.67 ± 0.11, respectively, both *p <* 0.001). The FIT2 program was inversely related to HbA_1c_ changes at 12 months (β = −1.33 ± 0.33, *p <* 0.001) while not related to HbA_1c_ changes at six months compared with usual care in the multiple linear regression analysis (Table S3).

In the time-varying analysis (*n* = 532), weight gain at six months was positively associated with HbA_1c_ changes at 6 and 12 months (β = 0.17 ± 0.05 and 0.51 ± 0.11, respectively, both *p <* 0.001), while post-program abstinence was only inversely related to HbA_1c_ changes at 12 months (β = −1.13 ± 0.34, *p <* 0.01) compared with the controls in the multiple linear regression analysis (Table S3).

### 3.3. New-Onset T2D and Regression to Normoglycemia

**Figure 1:**
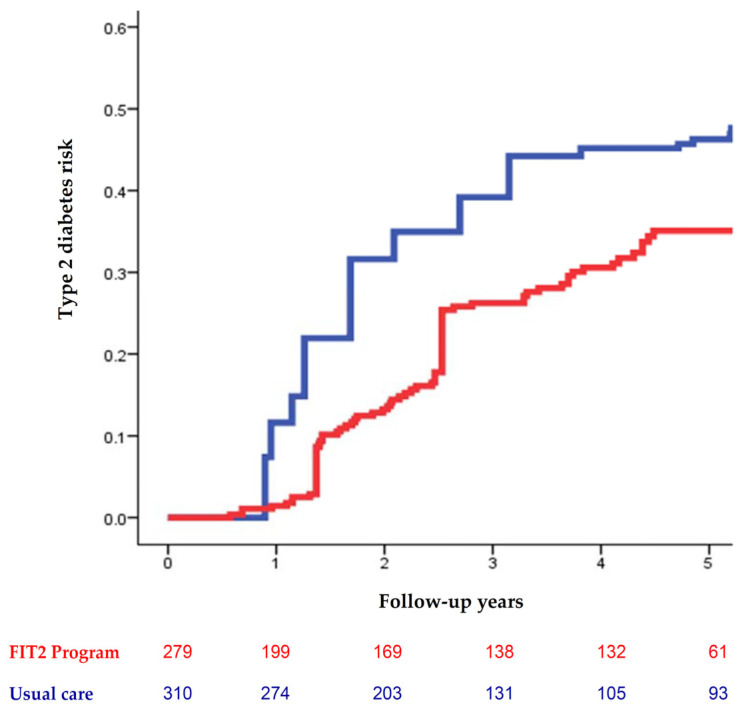
Long-term effect of the FIT2 program on type 2 diabetes risks in the intention-to-treat analysis.

From August 2013 through December 2020 (mean, 1316 days), 217 participants (36.8%) developed new-onset T2D, and 68 (11.5%) regressed to normoglycemia. In the intention-to-treat analysis, participants joining the FIT2 program group had a lower risk of incident T2D compared with the participants receiving usual care shown in the Kaplan–Meier failure plots (*p* = 0.008) (Figure 1). The FIT2 program was associated with a reduced risk of incident T2D (adjusted HR, 0.58; 95% CI, 0.40–0.84) and a higher probability of regression to normoglycemia (adjusted HR, 1.91; 95% CI, 1.04–3.53) compared with the usual care (Table 1). Weight gain at six months was positively associated with the T2D occurrence (adjusted HR, 1.19; 95% CI, 1.04–1.35) and negatively associated with regression to normoglycemia (adjusted HR, 0.72; 95% CI, 0.62–0.84). 

Kaplan–Meier failure plots show that participants joining the FIT2 program had a lower risk of incident type 2 diabetes (red line) compared with participants receiving usual care (blue line). The log-rank test was significant with *p* = 0.008.

**Figure 2:**
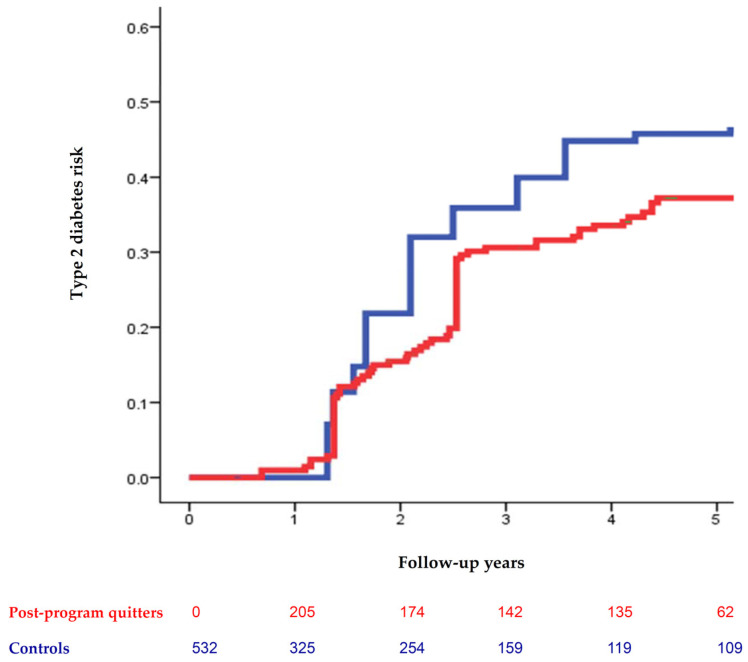
Long-term effect of the post-program abstinence on type 2 diabetes risk in the time-varying analysis.

In the time-varying analysis, the post-program quitters were at a lower risk of incident T2D than the controls revealed in the modified Kaplan–Meier failure plots (*p* = 0.016) (Figure 2). The post-program quitters had a lower T2D risk (adjusted HR, 0.63; 95% CI, 0.44–0.92) and were more likely to regress to normoglycemia (adjusted HR, 1.83; 95% CI, 1.01–3.30) compared with the controls (Table 2). Weight gain at six months was positively related to T2D risk (adjusted HR, 1.28; 95% CI, 1.13–1.45) and negatively related to the probability of regression to normoglycemia (adjusted HR, 0.73; 95% CI, 0.63–0.84).

Modified Kaplan–Meier failure plots show that the post-program quitters at six months had a lower risk of incident type 2 diabetes (red line) compared with the controls (blue line). The log-rank test was significant with *p* = 0.016.

Furthermore, participants with suspected depressive disorder had a higher incident T2D risk than those without, while physically active individuals were less likely to develop T2D than physically inactive individuals whether they were in the intention-to-treat or time-varying analyses (Table 1 and Table 2). However, neither suspected depressive disorder nor being physically active at baseline was associated with the probability of regression to normoglycemia. Among the post-program quitters (*n* = 207), an increased PCWG degree at six months was associated with an increased T2D risk (*p* for trend = 0.010) (Table S4). Post-program quitters with PCWG ≥ 2 kg at six months had a higher T2D risk compared with those with PCWG < 1 kg at six months (adjusted HR, 10.0; 95% CI, 2.11–47.5).

## 4. Discussion

This non-randomized controlled trial pioneered a shared decision-making based program combining smoking cessation therapy with individualized behavior coaching in diet and physical activity for PCWG restriction in prediabetic smokers. The intention-to-treat analysis demonstrated that the FIT2 program was negatively associated with long-term T2D risk and positively associated with the probability of regression to normoglycemia compared with usual care. The time-varying analysis consistently revealed that the post-program quitters had a lower long-term T2D risk and were more likely to regress to normoglycemia compared with the controls. In addition, weight gain at six months was positively associated with T2D occurrence and negatively associated with regression to normoglycemia in the both intention-to-treat and time-varying analyses. Among the post-program quitters, an increased PCWG degree at six months was associated with an increased T2D risk. Our integrated FIT2 program emphasizing both abstaining from smoking and restricting PCWG through individualized behavior coaching in diet and physical activity would reduce long-term T2D risk and increased the likelihood of regression to normoglycemia.

Although data from a nationally representative study showed that the mortality risk is smaller in overweight or obese ex-smokers than normal-weight smokers, primary care clinicians have been faced with challenges in fighting smoking and obesity together [8,21]. PCWG impairs glucose control through induced insulin resistance, increased appetite, and decreased energy expenditure, which offsets the long-term composite benefit of smoking cessation in prediabetes and diabetes patients [22,23,24]. Smoking was shown to lead to increased beta cell oxidative and endoplasmic reticulum stress, which would increase islet ceramide content, impair beta cell function, and reduce beta cell mass and proliferation [25]. Mice fed a high-fat diet and exposed to cigarette smoke for 11 weeks followed by an additional 11 weeks of no smoke exposure with a continued high-fat diet continued to experience defects in insulin processing and secretion [25]. Weight-management strategies should be implemented in smoking cessation programs. Nonetheless, a previous Cochrane review reported that most pharmacotherapies designed to reduce weight gain when stopping smoking lacked data on long-term efficacy [21].

The present study chose varenicline as the cessation medication, as it has been shown to excel in facilitating smoking cessation without evidence of an increased risk of neuropsychiatric or cardiovascular adverse events [26,27]. However, it is unclear whether varenicline affects weight control in the long term [21]. Moreover, whether administering varenicline for 16 weeks for smoking cessation in real practice impacts long-term changes in glucose homeostasis remains to be clarified [28]. Although we did not prescribe any anti-obesity medications in the present study, the average PCWG at six months in abstinent smokers (1.26 kg) was less than that reported in other observational studies evaluating weight gain from baseline (four to five kg) [24,29], demonstrating the realistic benefit of our individualized weight-management coaching in diet and physical activity. We also observed that the incident T2D risk was related to baseline physical activity and depressive disorder, which is consistent with the current literature [4,5,30,31]. This study emphasizes the real-practice effectiveness of an integrated program combining smoking cessation therapy with individualized behavior coaching in diet and physical activity for PCWG restriction regarding long-term glycemic outcomes. Although not all participants receiving the FIT2 program achieved post-program abstinence, the FIT2 participants were at a lower risk of HbA_1c_ elevation and new-onset T2D compared with participants receiving usual care. Furthermore, PCWG ≥ 2 kg at six months was associated with a much higher T2D risk than PCWG < 1 kg at six months in post-program quitters. Taken together, this study provides insights into conducting future multi-center clinical trials to test this integrated program on long-term cardiometabolic outcomes.

Some concerns and limitations of this study should be addressed. Immortal time bias is a challenge often overlooked in studies other than randomized controlled trials. It arises when the start of follow-up and treatment do not coincide [20]. In the time-varying analysis of the present study, we intended to evaluate the contribution of post-program abstinence; thus, the start of the treatment (post-program abstinence) was at six months after study entry by definition. To reduce immortal time bias in the survival analyses, all participants were considered unexposed from baseline until six months when the post-program abstinence status was established. Another issue is that of selection bias. We utilized a systematic identification system during screening and the general characteristics were comparable between the FIT2 program and the usual care participants in the intention-to-treat analysis at baseline. Additionally, we performed an intention-to-treat analysis among all participants joining the FIT2 program and receiving usual care to minimize selection bias from attrition. However, the selection bias could not be completely mitigated due to the allocation by shared decision making in this study. Participants with high motivation to quit were more likely to join the FIT2 program than their counterparts. Indeed, this design conforms to pragmatic clinical practice. The use of a non-randomized control group reduced the threat to external validity, which limits the value of randomized controlled trials. In this era of promoting a smoke-free environment, we advised smokers identified by the identification system to quit and assisted those willing to quit after assessment [15]. Preference-based allocation is the fundamental step in implementing shared decision-making [32].

## 5. Conclusions

Our integrated FIT2 program with the concerted concept of quitting smoking and restricting PCWG through individualized behavior coaching in diet and physical activity is negatively associated with the risk of new-onset T2D compared with usual care. Additionally, prediabetic smokers who successfully quit and maintained their non-smoking status at six months may achieve better long-term glycemic outcomes than the controls. More long-term health outcomes should be explored using shared decision-making-based study designs. To cut challenges in long-term T2D risk and increase the likelihood of regression to normoglycemia, we propose promoting organized tobacco cessation and weight control programs together for prediabetic smokers.

## Figures and Tables

**Table 1:** Factors for long-term glycemic outcomes among prediabetic smokers in the intention-to-treat analysis (*n* = 589).

	**New-Onset Type 2 Diabetes (217 Cases)**	
	**Unadjusted Analysis**	**Adjusted Analysis ^a^**
Body mass index (kg/m^2^)	1.05 (1.02–1.09) **	1.10 (1.05–1.16) ***
Weight gain at 6 months (kg)	1.17 (1.07–1.28) ***	1.19 (1.04–1.35) **
Physically active (vs. inactive)	0.29 (0.19–0.43) ***	0.31 (0.20–0.47) ***
Depression (vs. no)	3.58 (2.57–4.98) ***	2.34 (1.58–3.46) ***
FIT2 program (vs. usual care)	0.64 (0.48–0.84) **	0.58 (0.40–0.84) **
	**Regression to Normoglycemia (68 Cases)**	
	**Unadjusted Analysis**	**Adjusted Analysis ^a^**
Body mass index (kg/m^2^)	0.79 (0.73–0.86) ***	0.88 (0.79–0.97) *
Weight gain at 6 months (kg)	0.67 (0.58–0.77) ***	0.72 (0.62–0.84) ***
Physically active (vs. inactive)	1.76 (1.08–2.86) *	0.73 (0.43–1.23)
Depression (vs. no)	0.39 (0.12–1.26)	0.68 (0.19–2.37)
FIT2 program (vs. usual care)	1.85 (1.13–3.04) *	1.91 (1.04–3.53) *

Data are presented as hazard ratios (95% CIs). ^a^ Model: all listed clinical variables, age, sex, alanine aminotransferase, estimated glomerular filtration rate, hypertension, dyslipidemia, and sleep disturbance. * *p <* 0.05; ** *p <* 0.01; *** *p <* 0.001.

**Table 2:** Factors for long-term glycemic outcomes among prediabetic smokers in the time-varying analysis (*n* = 532).

	**New-Onset Type 2 Diabetes (217 Cases)**	
	**Unadjusted Analysis**	**Adjusted Analysis ^a^**
Body mass index (kg/m^2^)	1.05 (1.02–1.08) **	1.10 (1.05–1.15) ***
Weight gain at 6 months (kg)	1.20 (1.10–1.31) ***	1.28 (1.13–1.45) ***
Physically active (vs. inactive)	0.27 (0.18–0.42) ***	0.25 (0.15–0.39) ***
Depression (vs. no)	3.13 (2.25–4.36) ***	1.77 (1.19–2.64) **
Post-program quitters vs. controls	0.74 (0.56–0.97) *	0.63 (0.44–0.92) *
	**Regression to Normoglycemia (68 Cases)**	
	**Unadjusted Analysis**	**Adjusted Analysis ^a^**
Body mass index (kg/m^2^)	0.73 (0.65–0.82) ***	0.88 (0.79–0.97) **
Weight gain at 6 months (kg)	0.64 (0.55–0.73) ***	0.73 (0.63–0.84) ***
Physically active (vs. inactive)	1.80 (1.09–2.95) *	0.93 (0.53–1.63)
Depression (vs. no)	0.41 (0.15–1.15)	0.62 (0.20–1.93)
Post-program quitters vs. controls	1.67 (1.03–2.72) *	1.83 (1.01–3.30) *

Data are presented as hazard ratios (95% CIs). ^a^ Model: all listed clinical variables, age, sex, alanine aminotransferase, estimated glomerular filtration rate, hypertension, dyslipidemia, and sleep disturbance. *p < 0.05; ** *p <* 0.01; ***p < 0.001.

## Data Availability

The data presented in this study are available on request from the first author on reasonable request after full analyses of all secondary outcomes are reported.

## Supplementary Materials

The following are available online at https://www.mdpi.com/article/10.3390/nu13103360/s1, Figure S1: Study flowchart, Table S1: Baseline characteristics of participants (intention-to-treat analysis), Table S2: Characteristics of participants by different post-program abstinence statuses at six months (time-varying analysis), Table S3: Multiple linear regression analyses of HbA1c changes at 6 and 12 months in prediabetic smokers, Table S4: Association of post-cessation weight gain degree at 6 months with incident type 2 diabetes among post-program quitters (*n* = 207).
