# Supplementary material for: Incorporating Post-Cessation Weight-Control Coaching into Smoking Cessation Therapy to Reduce Type 2 Diabetes Risk
Source: Nutrients. 2021 Sep 25;13(10):3360. doi: 10.3390/nu13103360 (PMC8539112; doi:10.3390/nu13103360)
Supplement: Supplementary file 1 [file nutrients-13-03360-s001.zip › nutrients-1381338-supplementary.pdf]

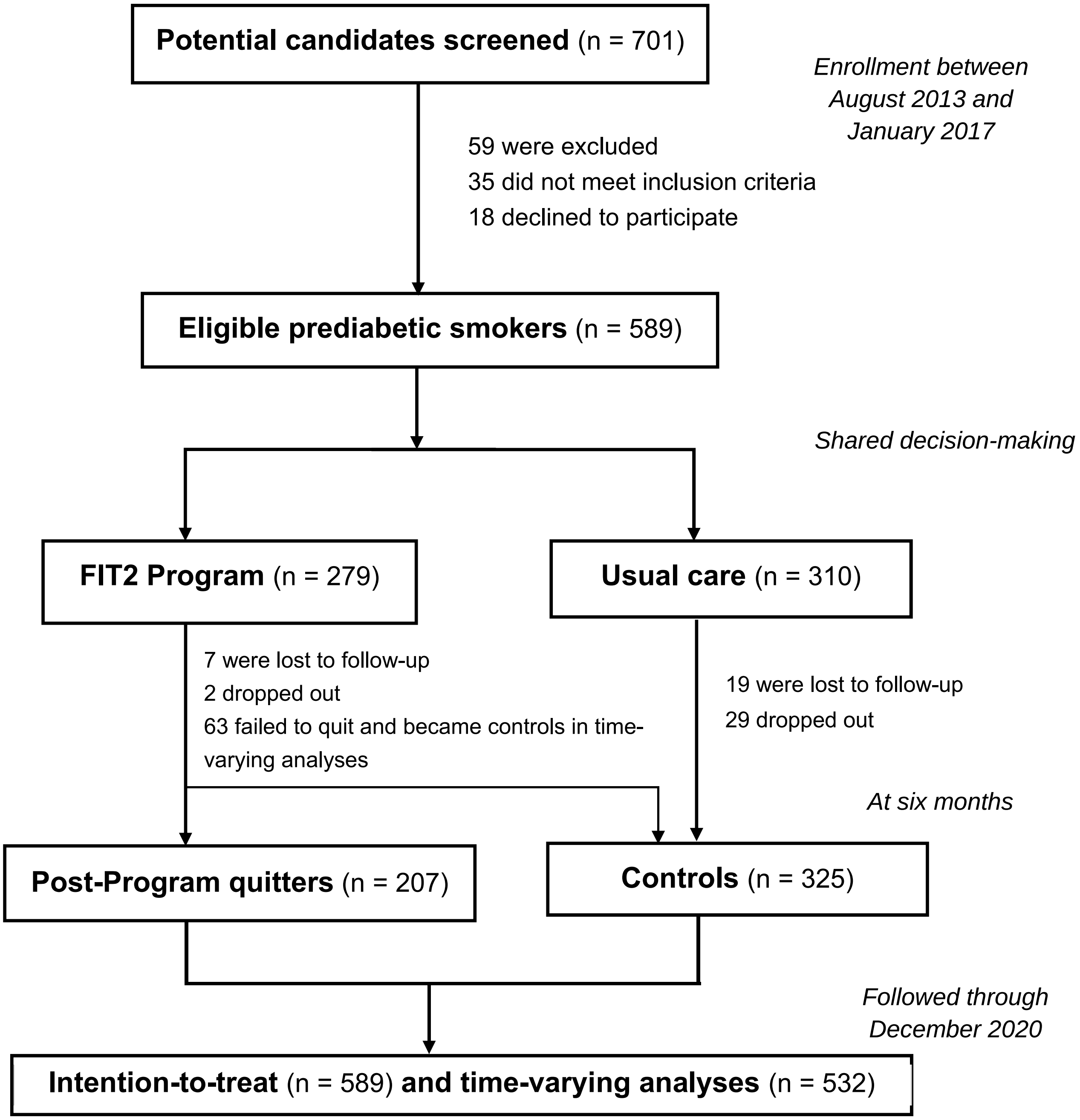

Figure S1. Study flowchart

The Fight Tobacco and Stay Fit (FIT2) program was a 16-week program including smoking cessation therapy with individualized behavior coaching in diet and physical activity for PCWG restriction. Post-program quitters were those who quit successfully after the intervention and maintained their non-smoking status at six months. The first six months experienced by all post-program quitters were considered time experienced by the controls in the time-varying analyses to reduce immortal time bias.

Table S1. Baseline characteristics of participants (intention-to-treat analysis)

	FIT2 program (n = 279)	Usual care (n = 310)	*P* value
Age	53.0 ± 10.5	53.9 ± 9.4	0.28
Male	232 (83.2)	259 (83.6)	0.90
Cigarette consumption (CPD)	18.4 ± 9.3	17.8 ± 8.2	0.39
Smoking duration (years)	27.5 ± 7.9	26.7 ± 7.8	0.18
FTND scores	5.76 ± 2.06	5.55 ± 2.03	0.20
Body mass index (kg/m^2)	26.0 ± 3.23	26.4 ± 3.91	0.17
HbA_{1c} (mmol/mol)	43.4 ± 1.6	43.3 ± 2.1	0.52
HbA_{1c} (%)	6.12 ± 0.14	6.11 ± 0.19	0.52
Fasting glucose (mmol/l)	6.16 ± 0.34	6.11 ± 0.39	0.13
ALT (IU/L)	46.7 ± 23.8	48.5 ± 29.1	0.41
eGFR (ml/min per 1.73 m^2)	87.1 ± 14.0	88.7 ± 11.8	0.15
Hypertension	161 (50.7)	157 (57.7)	0.09
Dyslipidemia	193 (69.2)	225 (72.6)	0.36
Physically active	82 (29.4)	73 (23.6)	0.11
Depression	26 (9.4)	29 (9.3)	0.99
Sleep disturbance	215 (77.1)	240 (77.4)	0.92

Data are presented as numbers (percentages) or means ± standard deviations.
ALT, alanine aminotransferase; CPD, cigarettes per day; FTND, Fagerström Test for Nicotine Dependence; GFR, estimated glomerular filtration rate.

Table S2. Characteristics of participants by different post-program abstinence statuses at six months (time-varying analysis)

	**Post-program quitters (n = 207)**	**Controls (n = 325)**	***P* value**
Age	53.6 ± 10.5	53.8 ± 9.4	0.86
Male	177 (85.5)	274 (84.3)	0.71
Cigarette consumption (CPD)	18.3 ± 9.6	17.9 ± 8.1	0.59
Smoking duration (years)	27.3 ± 8.3	26.6 ± 7.7	0.32
FTND scores	5.71 ± 2.11	5.58 ± 2.03	0.49
Body mass index (kg/m^2)	26.1 ± 2.8	26.5 ± 3.9	0.18
HbA_{1c} (mmol/mol)	43.3 ± 1.6	43.3 ± 2.1	0.96
HbA_{1c} (%)	6.11 ± 0.14	6.11 ± 0.19	0.96
Fasting glucose (mmol/l)	6.18 ± 0.35	6.11 ± 0.39	0.06
ALT (IU/L)	46.0 ± 23.8	48.5 ± 28.8	0.28
eGFR (ml/min per 1.73 m^2)	87.6 ± 13.5	88.5 ± 12.1	0.46
Hypertension	110 (53.1)	162 (49.9)	0.46
Dyslipidemia	145 (72.1)	242 (73.4)	0.27
Physically active	56 (27.1)	75 (23.1)	0.30
Depression	26 (12.6)	29 (8.9)	0.18
Sleep disturbance	157 (75.9)	250 (76.9)	0.78

Data are presented as numbers (percentages) or means ± standard deviations.
ALT, alanine aminotransferase; CPD, cigarettes per day; FTND, Fagerström Test for Nicotine Dependence; GFR, estimated glomerular filtration rate.

Table S3. Multiple linear regression analyses of HbA_{1c} changes at 6 and 12 months in prediabetic smokers

	**Intention-to-treat analysis** (n = 589)	
	HbA_{1c} change at 6 months	HbA_{1c} change at 12 months
Baseline HbA_{1c} (mmol/mol)	0.01 (0.04)	0.19 (0.09) *
Body mass index (kg/m^2)	0.05 (0.02) *	0.16 (0.05) ***
Weight gain at 6 months (kg)	0.24 (0.05) ***	0.67 (0.11) ***
Physically active (vs. inactive)	-0.21 (0.15)	-1.00 (0.31) **
Depression (vs. no)	0.38 (0.22)	0.02 (0.47)
FIT2 program (vs. usual care)	-0.10 (0.16)	-1.33 (0.33) ***
	**Time-varying analysis** (n = 532)	
	HbA_{1c} change at 6 months	HbA_{1c} change at 12 months
Baseline HbA_{1c} (mmol/mol)	0.05 (0.05)	0.29 (0.09) **
Body mass index (kg/m^2)	0.07 (0.02) **	0.18 (0.05) ***
Weight gain at 6 months (kg)	0.17 (0.05) **	0.51 (0.11) ***
Physically active (yes vs. inactive)	-0.15 (0.16)	-0.53 (0.32)
Depression (vs. no)	0.39 (0.22)	0.13 (0.45)
Post-program abstinence (vs. control)	-0.03 (0.17)	-1.13 (0.34) **

Data are presented as β (SE), adjusted for age, sex, alanine aminotransferase, estimated glomerular filtration rate, hypertension, dyslipidemia, and sleep disturbance. $^{*}P<0.05$; $^{**}P<0.01$; $^{***}P<0.001$.

Table S4. Association of post-cessation weight gain degree at 6 months with incident type 2 diabetes among post-program quitters (n=207)

	**Unadjusted analysis**	**Adjusted analysis** [a]
PCWG <1 kg (n=69)	Referent	Referent
PCWG 1 to 2 kg (n=57)	2.85 (0.74–11.0)	1.36 (0.31–5.97)
PCWG ≥2 kg (n=81)	31.5 (9.83–101) **	10.0 (2.11–47.5) *
P for trend	<0.0001	0.010

Data are presented as hazard ratios (95% CIs). [a] Adjusted for age, sex, body mass index, post-program abstinence, alanine aminotransferase, estimated glomerular filtration rate, hypertension, dyslipidemia, physical activity, depression, and sleep disturbance. PCWG, post-cessation weight gain. *$P<0.05$; ** $P<0.01$.
